# Risk Factors Associated with Overweight and Obesity in Japanese-Brazilians

**DOI:** 10.1155/2018/5756726

**Published:** 2018-05-08

**Authors:** Ivi R. Back, Rosana R. Oliveira, Eraldo S. Silva, Sonia S. Marcon

**Affiliations:** ^1^Program in Health Sciences, State University of Maringá, 87.020-900 Maringá, PR, Brazil; ^2^Department of Nursing, State University of Maringá, 87.020-900 Maringá, PR, Brazil; ^3^Program in Biostatistics, State University of Maringá, 87.020-900 Maringá, PR, Brazil

## Abstract

**Objective:**

To estimate which risk factors (sociodemographic, lifestyle, and health conditions) are associated with overweight and obesity in Japanese-Brazilians.

**Methods:**

This was a cross-sectional study carried out with Japanese-Brazilians living in the southern region of Brazil. Data were collected between March and December of 2016 through a household survey addressing sociodemographic characteristics, lifestyle, and health conditions. Data were analyzed by means of logistic regression considering 95% level of significance.

**Results:**

A total of 542 Japanese-Brazilians with a mean age of 47.75 years were evaluated; 52.8% were eutrophic, 36.9% were overweight, and 10.3% were people with obesity. The following variables remained associated with overweight after adjustments; male gender (ORaj = 1.85, CI = 1.24–2.76), age range of 40–49 years (ORaj = 2.27, CI = 1.10–4.68), and 50 to 59 years (ORaj = 2.17, CI = 1.004–4.72), alcohol consumption (ORaj = 2.11, CI = 1.07–4.16), and presence of chronic disease (ORaj = 1.59, CI = 1.02–2.46). The following were independent factors associated with obesity: male gender (ORaj = 3.63, CI = 1.78–7.40), the presence of chronic disease (ORaj = 4.13, CI = 1.96–8.71), the age range of 30 to 39 years (ORaj = 4.74, CI = 1.65–13.64) and 40 to 49 years (ORaj = 2.89, CI = 1.05–7.95), and irregularly active lifestyle (ORaj = 2.73, CI = 1.12–6.69).

**Conclusion:**

The results of this study show that being a male in the age range of 30–49 years old, alcohol consumption, and presence of chronic disease are associated with overweight and obesity in Japanese-Brazilians.

## 1. Introduction

Overweight has become a serious public health problem in many countries and is associated with chronic diseases such as metabolic syndrome, hypertension, and diabetes mellitus [1]. The increase in the number of adults who were overweight or with obesity (body mass index-BMI ≥25 kg/m^2^) is alarming. The prevalence of obesity has doubled in more than 70 countries since 1980; 603.7 million adults worldwide will be with obesity by 2015 [2].

Thus, identifying the prevalence and factors associated with overweight and obesity has become the goal of many studies [1–4] since the obesity epidemic has been expanding globally. A study conducted in 31 countries including 20 in Europe, 8 in Asia, and Australia, Chile, and the USA reports the prevalence of overweight individuals and with obesity of 31.7% and 12.4%, respectively [3].

Although obesity is a chronic and multifactorial disease, genetic and environmental factors must also be considered, which are reflected in specific populations with high or low rates [3, 5] as well as in some ethnic groups that denote more or fewer risks [5].

The National Nutrition Survey in Japan conducted in 2015 showed 18.9% overweight prevalence in women and 28% in men (BMI >25 kg/m^2^). In Brazil, studies have found higher rates of overweight and obesity in Japanese-Brazilians than those in Japan [4, 6, 7]. Overweight ranged from 34.5% in Curitiba in Paraná State [4] to 45.63% in Bauru in São Paulo State [7]. Another more specific study reported that out of the 1,581 Japanese-Brazilians evaluated in Bauru, 36.2% were overweight and 8.7% were already with obesity [6].

In addition to the prevalence, the knowledge about risk factors that may be associated with overweight and obesity can subsidize the implementation of measures aimed at sensitizing this population about the importance of some behaviors and of prophylactic and sensitization procedures. Awareness about these associations can be useful to prevent overweight and obesity; these associations are still not clear and well-delimited.

Therefore, the objective of this study was to estimate the risk factors associated with overweight and obesity in Japanese-Brazilians.

## 2. Materials and Methods

### 2.1. Participants x Sampling

This was a cross-sectional study using a household survey conducted in the city of Maringá in Paraná State, where one of the largest Japanese immigrant descents communities lives in Brazil. The study included Japanese-Brazilians of both genders and age equal to or older than 18 years and excluded individuals with physical disabilities and pregnant and breastfeeding women.

The Nikkei Census conducted in the municipality between 2008 and 2009 was used to calculate the sample size. This census identified a population of 14,324 Japanese-Brazilians; individuals; prevalence of 0.4% a overweight in the study population [4, 7]; with 95% confidence level (*z* = 1.96) and a sample error of 4% (*e* = 0.04) resulting in a sample size calculated as 576, which added by 5% for possible losses resulted in a total of 603 individuals.

The selection of participants was based on information from the Nikkei Census, which showed similarities between the urban and living conditions of Japanese-Brazilians (housing, infrastructure, sanitation, education, health services, and educational level). This allowed grouping neighborhoods in seven continuous regions (stratum). Subsequently, these participants were randomly picked, proportionally to the total number of Japanese-Brazilians residents in each stratum.

Those who were drawn were located based on the addresses listed in the Nikkei census or indication from other Japanese-Brazilian residents in the same stratum. Those not located after three visits conducted on different days and different times, as well as those who refused to participate in the study, were replaced by individuals residing in the same stratum in order to maintain the total sample and stratum sizes. It is noteworthy that a total of 150 replacements were performed and that subjects with low weight (*n*=61) were excluded from the present analysis.

### 2.2. Procedures and Study Variables

The data were collected from March to December of 2016 through a semistructured interview and the use of a questionnaire addressing sociodemographic characteristics, lifestyle, and health conditions. The sociodemographic variables were gender, generation of immigrants (issei: Japanese immigrants; nisei: children of immigrants; sansei: grandchildren of immigrants; and yonsei: great grandchildren of immigrants) [8], age, marital status (with partner/without partner), educational level (illiterate; elementary/incomplete middle; middle/incomplete high school; high school/incomplete college; college), and purchasing power. Purchasing pocalculation of the Brazilian Association of Companies and Research–ABEP [9].

The variables related to lifestyle were (a) self-referred diet: good, fair, and poor as proposed in the Ministry of Health's Food Guide [10]; (b) daily consumption of fruits: consumes—three or more portions, unsatisfactory—one or two portions, no consumption; (c) daily consumption of vegetables: consumes—three or more portions, unsatisfactory—one or two portions, no consumption; (d) alcohol consumption: no consumption, low-risk consumption, and risky consumption [11]; (e) smoking: classified as low level, elevated, and nonsmoking [12], and (f) practice of physical activity: active, irregularly active, and sedentary [13].

The evaluation of health conditions included: (a) self-perception of health: classified as excellent, very good, good, fair, and poor; (b) the presence of chronic autoreferred diseases: yes/no. At the time of the interview, the blood pressure was verified in a *Premium*® aneroid pressure device, with a cuff suitable for the brachial perimeter, individual sitting, uncrossed legs, feet resting on the floor, right arm resting on the heart, after three to five minutes of rest. At least two checks were performed, with an interval of one minute between them. A third measurement was performed when the first two were very different. The mean of the two measurements was considered to be the nearest. The blood pressure value was classified as altered when systolic blood pressure (SBP) ≥140 and/or diastolic blood pressure (DBP) ≥90 [14].

Weight and height were measured for the evaluation of the nutritional status according to age: (a) ≥60 years: low weight (<22 kg/m^2^), eutrophic (22–27 kg/m^2^), and overweight (27 kg/m^2^) [15]; (b) ≥20–≤59 years: low weight (<18.5 kg/m^2^); eutrophic (18.5–24.9 kg/m^2^); overweight (25–29.9 kg/m^2^); and obese (>30 kg/m^2^) [16]; (c) ≤19 years: low weight (percentile 3); eutrophic (percentile ≥3–<percentile 85); overweight (≥percent 85–<percentile 97); obese (≥percentile 97) [17, 18].

### 2.3. Statistical Analysis

Data were entered and organized in Microsoft Excel worksheets and later analyzed using the Statistical Analysis Software (SAS, Version 9.3).

Univariate logistic regression was performed to verify the risks for overweight and obesity in relation to eutrophy. All variables with *p* value < 0.20 in the univariate analysis were included in the multiple logistic regression analysis. Those with *p* value < 0.05 remained in the final model; variables that acted as adjustments were identified. A significance level of 95% was considered.

### 2.4. Ethical Precepts

All participants were informed about the objectives of the study and type of participation desired and signed the free and informed consent term. The project was approved by the Committee on Ethics in Research with Human Beings of the Maringá State University (Opinion *n*° 1 150 115/2015).

## 3. Results

A total of 542 Japanese-Brazilians aged 18–87 years (mean age 47.75 years ±18.14) were selected for this study; 52.8% were eutrophic, 36.9% were overweight, and 10.3% were with obesity.

Most of the women presented eutrophy (62.5%), 30.6% were overweight, and 6.9% were with obesity. A large portion of men was overweight (44.1%), 41.7% were eutrophic, and 14.2% were with obesity.

Thus, in relation to women, men presented a significant risk of being overweight (ODDS: 2.161, CI: 1.49–3.12; *p* < 0.001) and with obesity (ODDS: 3,057; CI: 1.68–5.55; *p* < 0.001) compared to the eutrophic condition; this risk was double for overweight and triple for obesity.

The odds of being overweight and with obesity were significantly higher as age increased; the chance of being overweight was three times higher in the age range between 30 and 49 years (*p* < 0.001) and almost five times higher (*p* < 0.001) in the age range between 50 and 59 years compared to the other age ranges. The odds of individuals with partners of being overweight were almost twice higher than individuals without partners (*p* < 0.001).

Regarding professional activities, self-employed individuals showed a 3.60-fold higher chance of being with obesity (*p*=0.001) than those in other professions; being a student was a protective factor for the overweight condition (*p*=0.01).  Table 1 shows other associations between overweight and obesity and sociodemographic variables.

Table 2 shows that the chance of irregularly active individuals presenting obesity was almost twice as high as those with sedentary behavior among eutrophics. The odds of being overweight and obesity were respectively three and four times higher than those with sedentary behavior among those who consumed alcohol. Those with poor eating habits were three times more likely to be with obesity compared to those in other groups. Likewise, those who did not consume or had unsatisfactory consumption of fruits were at least three times more likely to be with obesity compared to those who consumed fruits.

The distribution of health conditions among participants was 15.3% diabetes mellitus, 46.7% hypertension, 23.6% hypercholesterolemia, 3.1% kidney problems, 3.7% cancer, 4.4% cardiovascular disease, and 1.5% stroke. Those with self-reported chronic diseases (diabetes mellitus, hypertension, hypercholesterolemia, cancer, kidney and heart issues, and stroke) showed chances for being overweight and obesity increased by almost two-fold compared to eutrophic individuals (Table 2). It was also observed that presenting altered blood pressure doubles the chances of being overweight, and quintuple the chances of being with obesity.

The multivariate logistic regression analysis for being overweight after adjusting for marital status showed that being a male between 40 and 59 years old and consuming alcoholic beverages almost doubles the chance of being overweight. Moreover, the presence of some type of chronic disease was associated with being overweight (Table 3).

Conversely, the multivariate logistic regression analysis for obesity demonstrated that being male or presenting some type of chronic disease increases the chance of obesity by almost four times. The age group of young adults between 30 and 49 years of age presented associations with obesity. Regarding the practice of physical activity, being irregularly active almost tripled the chances of obesity (Table 3).

## 4. Discussion

The prevalence of overweight individuals in immigrants is already the focus of several studies. However, data on associations between some risk factors such as lifestyle, health conditions, and sociodemographic characteristics and overweight and obesity in Japanese-Brazilians are still scarce. In this study, a significant association was observed after adjustments between overweight and obesity in young male adults (between 30 and 59 years) and the presence of chronic diseases. Moreover, individuals who consume alcoholic beverages are more likely to be overweight, and those who irregularly practice physical activities showed increased risks for obesity.

Population studies show that the incidence of overweight and obesity is increasing worldwide [1–3]. Japanese-Brazilians are probably included in this scenario as a consequence of Westernization in their life habits [4]; some studies with this specific population [4, 6, 19] report high rates of overweight and obesity corroborating the process of acculturation.

In Brazil, the prevalence of overweight increased 11.2% in 10 years, from 42.6% in 2006 to 53.8% in 2016 [20], while in Japan, the prevalence of overweight/obesity in 2015 was 23.1% [21]. The prevalence of overweight (overweight 36.9% and obesity 10.3%) identified in this study is closer to the prevalence in Brazil (53.8%) than that in Japan (23.1%) suggesting the influence of the process of acculturation to which immigrants are exposed.

The sociodemographic characteristics of the studied population show that men are almost twice as likely to be overweight (ORaj: 1.85, CI: 1.24–2.76), and almost four times more likely to be with obesity than women (ORaj: 3.63; CI: 1.78–7.40). These results reinforce results of other studies with Japanese-Brazilians [4, 22]. A study carried out in Bauru with 772 Japanese-Brazilians observes that the intake of fat and processed food were positively associated with obesity in men [19]. Thus, men are probably more vulnerable to risk factors to being overweight, which points to the need to broaden and deepen the gaze on gender differences to support adequate planning for coping and treatment of health conditions associated with excess weight.

The odds of being overweight are higher among individuals aged 40 to 49 years (ORaj: 2.27, CI: 1.10–4.68) and 50 to 59 years (ORaj: 2.17; IC: 1.004–4.72) than at other age ranges. The odds of obesity are higher among individuals aged 30 to 39 years (ORaj: 4.74, CI: 1.65–13.64) and 40 to 49 (ORA: 2.89, CI: 1.05–7.95) than at other age ranges. These results differ from those reported in a study with a Spanish population including 21,007 adults reporting that the risk of overweight and obesity is higher in individuals aged over 74 years than in younger individuals [1]. Considering the differences between these two populations with regard to age structure and living conditions, which influence life expectancy, it is worth noting that overweight appears in that Spanish population in later decades compared to the studied Japanese-Brazilian population. Other studies with Japanese-Brazilians also report high overweight and obesity prevalence in young adults [7, 23]. Therefore, this population needs to be sensitized and closely monitored by the health sector because individuals with obesity are three to four times more likely to develop cardiometabolic problems compared to those at normal weight [24].

Our results show that individuals with chronic diseases are almost twice as likely to be overweight (ORaj: 1.59; CI: 1.02–2.46) and four times as likely to be with obesity (ORaj: 4, 13, CI: 1.96–8.71) than individuals without chronic diseases. A study carried out in Bauru in Brazil with 1,030 Japanese-Brazilians shows high rates of chronic diseases distributed as 45.4% hypertension, 34.9% diabetes mellitus, 62.2% hypercholesterolemia, 64.3% increased triglycerides, and 21.1% peripheral arterial obstructive disease [22]. Those authors suggest that these results reflect the exposure to a westernized environment, which exacerbates the genetic tendency to accumulate body fat. The different results observed in the present study (15.3% diabetes mellitus, 46.7% hypertension, and 23.6% hypercholesterolemia) might be due to the use of different methodologies to diagnose these chronic diseases; these diseases were self-reported in this study and diagnosed through laboratory exams in that study.

A significant increase in the prevalence of chronic diseases has been observed in Brazil in the last decade (61.8% of diabetes and 14.2% of hypertension) [20]. Despite the increased prevalence among Brazilians, the tendency to present chronic diseases in the studied population is similar to that reported in previous studies with Japanese-Brazilians [6, 22, 25]. Therefore, it is important to promote awareness among immigrant populations in maintaining a normal weight considering the number of benefits to the nondevelopment of chronic diseases. According to a study carried out in 195 countries with data from 68.5 million people in 2015, high BMI contributed to 4.0 million deaths [2].

In this sample, both low-risk consumption (ORaj: 1.417; CI: 0.93–2.14) and high-risk consumption of alcoholic beverages were associated with overweight (ORaj: 2.11; CI: 1.07–4.16). A population-based study including 48,741,000 adults also observed an association between alcohol consumption and overweight; the study reports that alcohol consumption (several times a month) was associated with overweight in 31 evaluated countries (ORaj: 1.13 CI: 1.04–1.22) [3]. Therefore, the present study helps to clarify the complex relationship between alcohol consumption and obesity, particularly in the context of lifestyle behaviors and health conditions [26].

Individuals who practice physical activity irregularly in terms of frequency or duration are three times more likely to be with obesity (ORaj: 2.73; CI: 1.12–6.69). These findings differ from those reported in a study about behavioral risk factors performed in 31 countries reporting that physical inactivity was inversely associated with overweight but not associated with obesity [3]. Such findings are probably explained by the difference in criteria used to classify individuals as physically active or inactive. That study considered individuals who performed at least 20 minutes of daily, several times a week, or sometimes in a month of physical activity enough to break a sweat or breathe deeply as active. Our study considered the frequency (number of minutes performing a physical activity) and days (number of days per week) to estimate minutes\week of physical activity considering >150 minutes/week as the cutoff point to be classified as physically active. Physical activity contributes to energy consumption, prevents obesity, favors weight loss, and reduces the risk of developing cardiovascular diseases, type 2 diabetes mellitus, and some types of cancer [27].

It is important to emphasize that the design of this cross-sectional study does not allow establishing a cause-effect relationship. However, the results increase the knowledge about the risk factors associated with the conditions of overweight and obesity in Japanese-Brazilians. Hence, considering the small number of studies on factors associated with overweight and obesity in Japanese-Brazilians, it is believed that our results can support the implementation of interventions aimed at weight control and consequently prevention of related diseases in this population.

## 5. Conclusions

The results of this study allow us to affirm that being male, age (30–49 years), alcohol consumption, and the presence of chronic disease are associated with overweight and obesity in Japanese-Brazilians. Thus, better planning and implementation of care would contribute to nutritional behavior and decrease the risk factors associated with overweight and obesity in this population.

## Figures and Tables

**Table 1:** Risk of overweight and obesity related to eutrophy in sociodemographic variables of Japanese-Brazilian individuals. Maringá-PR, Brazil, 2016.

BMI–Brazil									
Variables	Eutrophic *n* (%) 286	Overweight *n* (%) 200	Odds ratio	CI (95%)	*p* value	With obesity *n* (%) 56	Odds ratio	CI (95%)	*p* value
Gender									
Female	180 (62.94)	88 (44.00)		—		20 (35.71)		—	
Male	106 (37.06)	112 (56.00)	2.161	1.49–3.12	<0.0001	36 (64.29)	3.057	1.68–5.55	<0.0001
Generation									
Isei	10 (3.50)	3 (1.50)		—		0		—	
Nisei	128 (44.76)	98 (49.00)	2.55	0.68–9.52	0.163	16 (28.57)	>999	<0.001–999	0.957
Sansei	121 (42.31)	90 (45.00)	2.47	0.66–9.26	0.177	36 (64.29)	>999	<0.001–999	0.953
Yonsei	27 (9.44)	9 (4.50)	1.11	0.24–4.95	0.890	4 (7.14)	>999	<0.001–999	0.956
Age range									
1 to 29 years	82 (28.67)	31 (15.50)		—		8 (14.29)		—	
30 to 39 years	30 (10.49)	19 (9.50)	1.67	0.82–3.40	0.15	16 (28.57)	5.46	2.12–14.08	<0.0001
40 to 49 years	40 (13.99)	46 (23.00)	3.04	1.68–5.49	<0.001	15 (26.79)	3.84	1.50–9.81	0.005
50 to 59 years	35 (12.24)	40 (20.00)	3.02	1.63–5.58	<0.001	17 (30.36)	4.97	1.96–12.60	0.001
60 or +	99 (34.62)	64 (32.00)	1.71	1.01–2.87	0.04	0	0.00	0.000	0.99
Financial status									
A	77 (26.92)	66 (33.00)		—		12 (21.43)		—	
B1	86 (30.07)	60 (30.00)	0.81	0.51–1.29	0.386	19 (33.93)	1.417	0.64–3.10	0.383
B2	98 (34.27)	63 (31.50)	0.75	0.47–1.18	0.216	19 (33.93)	1.244	0.56–2.71	0.584
C1	16 (5.59)	10 (5.00)	0.72	0.31–1.71	0.469	4 (7.14)	1.604	0.45–5.61	0.459
C2-D	9 (3.15)	1 (0.50)	0.13	0.01–1.05	0.05	2 (3.57)	1.426	0.27–7.41	0.673
Education									
Illiterate	8 (2.80)	1 (0.50)	0.15	0.01–1.23	3.116	3 (5.36)	2.054	0.50–8.37	0.315
Elementary/incomplete middle	27 (9.44)	19 (9.50)	0.85	0.44–1.62	0.236	0	<0.001	<0.001–999	0.968
Middle/incomplete high school	32 (11.19)	19 (9.50)	0.71	0.38–1.34	1.057	3 (5.36)	0.513	0.14–1.83	0.304
High school/incomplete college	104 (36.36)	66 (33.00)	0.76	0.50–1.15	1.580	29 (51.79)	1.52	0.82–2.84	0.181
College	115 (40.21)	95 (47.50)		—		21 (37.50)		—	
Marital status									
With a partner	156 (54.55)	140 (70.00)	1.94	1.32–2.84	0.001	36 (64.29)	1.500	0.82–2.71	0.18
Without a partner	130 (45.45)	60 (30.00)		—		20 (35.71)		—	
Professional status									
Retired	61 (21.3)	39 (19.50)	0.84	0.51–1.39	0.50	2 (3.60)	0.23	0.51–1.05	0.05
Self-employed	53 (18.5)	56 (28.00)	1.39	0.86–2.25	0.17	27 (48.20)	3.60	1.74–7.45	0.001
Home maker	26 (9.1)	15 (7.5)	0.76	0.37–1.53	0.44	7 (12.50)	1.90	0.69–5.20	0.20
Working	99 (34.6)	75 (37.5)		—		14 (25.00)		—	
Student	47 (16.4)	15 (7.5)	0.42	0.21–0.81	0.01	6 (10.70)	0.90	0.32–2.49	0.84

**Table 2:** Risk of overweight and obesity related to eutrophy and lifestyle and health conditions of Japanese-Brazilians in the city of Maringá-PR, Brazil, 2016.

Variables	Eutrophic *n* (%)	Overweight *n* (%)	Odds ratio	CI (95%)	*p* value	With obesity *n* (%)	Odds ratio	CI (95%)	*p* value
*Lifestyle*									
Tobacco use									
Low	4 (1.40)	6 (3.00)	2.21	0.61–7.96	0.22	5 (8.93)	6.95	1.80–26.77	0.005
Elevated	4 (1.40)	6 (3.00)	2.21	0.61–7.96	0.22	1 (1.79)	1.39	0.15–12.69	0.77
Nonsmoker	278 (97.20)	188 (94.0)		—		50 (89.29)		—	
Physical activity									
Active	77 (26.92)	53 (26.50)		—		11 (19.64)		—	
Irregularly active	120 (41.96)	92 (46.00)	1.11	0.71–1.73	0.63	34 (60.71)	1.98	0.94–4.14	0.06
Sedentary	89 (31.12)	55 (27.50)	0.89	0.55–1.45	0.63	11 (19.64)	0.86	0.35–2.10	0.74
Alcohol consumption									
Does not consume	176 (61.5)	95 (47.5)		—		24 (42.9)		—	
Low-risk consumption	92 (32.2)	74 (37.0)	1.49	1.00–2.21	0.048	22 (39.3)	1.74	0.93–3.29	0.081
Risky consumption	18 (6.3)	31 (15.5)	3.19	1.69–6.00	0.000	10 (17.9)	4.07	1.68–9.85	0.002
Eating habits									
Regular	201 (70.28)	148 (74.00)	1.32	0.83–2.10	0.23	37 (66.07)	1.28	0.59–2.81	0.524
Bad	22 (7.69)	17 (8.50)	1.39	0.65–2.96	0.39	10 (17.86)	3.18	1.14–8.85	0.026
Good	63 (22.03)	35 (17.50)		—		9 (16.07)		—	
Fruit consumption									
Consumer	87 (30.4)	59 (29.50)		—		6 (10.7)		—	
Not satisfactory	151 (62.8)	112 (56.00)	1.09	0.72–1.65	0.66	37 (23.2)	3.55	1.44–8.75	0.006
Does not consume	48 (16.8)	29 (14.5)	0.89	0.50–1.57	0.69	13 (16.4)	3.92	1.40–10.99	0.009
Vegetable consumption									
Consumer	228 (79.7)	166 (83.00)		—		42 (75.00)		—	
Not satisfactory	49 (17.10)	23 (11.50)	0.10	0.37–1.10	0.64	10 (17.90)	1.10	0.52–2.35	0.79
Does not consume	9 (3.10)	11 (5.50)	0.26	0.68–4.14	0.68	4 (7.10)	2.41	0.71–8.19	0.15
*Health conditions*									
Blood pressure alteration									
Altered	100 (34.97)	112 (56.0)	2.36	1.63–3.42	<0.001	41 (73.21)	5.08	2.68–9.63	<0.001
Not altered	186 (65.03)	88 (44.00)		—		15 (26.79)		—	
Chronic disease^∗^ Self-referred									
No	174 (60.84)	95 (47.50)		—		24 (42.86)		—	
Yes	112 (39.16)	105 (52.50)	1.72	1.19–2.47	0.004	32 (57.14)	2.07	1.16–3.70	0.014
Health self-perception									
Excellent	43 (15.00)	33 (16.50)		—		7 (12.50)		—	
Very good	73 (25.50)	33 (16.50)	0.62	0.33–1.15	0.13	6 (10.70)	0.50	0.15–1.60	0.24
Good	133 (46.50)	93 (46.50)	0.94	0.55–1.59	0.94	30 (53.60)	1.38	0.56–3.38	0.47
Regular	34 (11.90)	35 (17.50)	1.38	0.71–2.66	1.38	8 (14.30)	1.44	0.47–4.38	0.51
Bad	3 (1.00)	6 (3.00)	2.68	0.62–11.56	2.68	5 (8.90)	10.23	1.98–52.73	0.005

^∗^The following were considered chronic diseases: diabetes, hypertension, hypercholesterolemia, cancer, and kidney and heart problems.

**Table 3:** Logistic regression of factors associated with overweight and obesity in Japanese-Brazilians living in Maringá-PR, Brazil, 2016.

Variables	Odds ratio (ORaj)	CI (95%)	*p* value
*Overweight* ^∗^			
Gender			
Female	—	—	—
Male	1.857	1.24–2.76	0.002
Age range			
1 to 29 years	—	—	
30 to 39 years	1.397	0.65–2.99	0.389
40 to 49 years	2.275	1.10–4.68	0.026
50 to 59 years	2.177	1.004–4.72	0.049
60 or +	1.128	0.56–2.25	0.734
Marital status			
With a partner	1.162	0.69–1.93	0.563
Without a partner	—	—	—
Alcohol consumption			
Does not consume	—	—	—
Low-risk consumption	1.417	0.93–2.14	0.099
Risky consumption	2.110	1.07–4.16	0.031
Chronic disease^#^ (self-referred)			
No	—	—	—
Yes	1.592	1.02–2.46	0.037
*With obesity*			
Gender			
Female	—	—	—
Male	3.633	1.78–7.40	<0.001
Chronic disease			
Yes	4.139	1.96–8.71	<0.001
No	—	—	—
Age range			
1 to 29 years	—	—	—
30 to 39 years	4.746	1.65–13.64	0.004
40 to 49 years	2.898	1.05–7.95	0.039
50 to 59 years	2.335	0.79–6.84	0.122
60 or +	0.000	0.000	0.996
Physical activity			
Active	—	—	—
Irregularly active	2.737	1.12–6.69	0.027
Sedentary	0.851	0.30–2.36	0.757

^∗^Model adjusted for marital status and low-risk alcohol consumption. ^#^The following were considered chronic diseases: diabetes, hypertension, hypercholesterolemia, cancer, and kidney and heart problems.

## Data Availability

The data used to support the findings of this study were provided under license, access to these data will be considered by any the authors and may be requested from the corresponding author.
